# The effect of smoking on the marginal bone loss around implant-supported prostheses

**DOI:** 10.18332/tid/109279

**Published:** 2019-05-20

**Authors:** Emre Mumcu, Arzu Beklen

**Affiliations:** 1 Department of Prosthodontics, Faculty of Dentistry, Osmangazi University, Eskisehir, Turkey; 2 Department of Periodontology, Faculty of Dentistry, Osmangazi University, Eskisehir, Turkey

**Keywords:** smoking, marginal bone loss, implant, fixed prostheses, removable prostheses

## Abstract

**INTRODUCTION:**

Implantology has led to several changes in the planning process involved in the application of dental prostheses to diminish bone level changes along the margins of dental implants. However, the relationship between smoking and marginal bone loss around dental implants, supporting both fixed and removable prostheses has not been investigated. We hypothesize that the design of different prostheses alter the effects of smoking, which consequently affects the amount of supporting alveolar bone.

**METHODS:**

In this study, we included 137 implants in the ‘implant-supported fixed prostheses’ (ISFP) group (31 smokers, 106 non-smokers) and 94 implants (21 smokers, 73 non-smokers) in the ‘implant-supported removable prostheses’ (ISRP) group. The corresponding patients were examined in routine recall sessions conducted at 6, 12 and 24 months after the placement of the dental prostheses. The recorded clinical periodontal parameters were the presence/ absence of a plaque index, bleeding index, and the probing depths. These periodontal parameters were assessed in conjunction with marginal bone level measurements. Comparative bone level measurements were obtained from radiographical images at ×20 magnification using the CorelDraw 11.0 software program. Statistical analysis was performed using the SPSS Statistical Software version 21.0.

**RESULTS:**

The overall clinical parameters were found to be poorer in smokers than in non-smokers (p<0.05). In all the groups, time-dependent bone loss was observed. However, among the patients with ISRPs, smokers were associated with significantly greater marginal bone loss compared to patients with ISFPs (p<0.05).

**CONCLUSIONS:**

In smokers with dental ISRPs, the marginal bone loss rates are likely to reach critical levels. Therefore, after the placement of prostheses, strict recall periods with a dental professional should be observed, and their guidance should be implemented in order to monitor the health of the bones around the implants.

## INTRODUCTION

Tooth loss can cause the appearance of an incomplete smile or functional disability, which affects the life quality of patients^1^. Fortunately, osseointegrated implants and modern prosthetic applications closely mimic the missing teeth and their function. A successful osseointegration can be defined as the direct structural and functional connection of an implant with the surrounding bone; this procedure has an overall success rate of approximately 95% despite the biological complications that might still occur^2^.

Dental implants lack the periodontal ligament compared to natural teeth. Otherwise, the soft tissue surrounding implants has a sulcular region very similar to a tooth. The epithelial cells of the sulcular region are supported by a layer of connective tissue above the bone^3^. The stability of these surrounding tissues is influenced by dynamic processes involving cellular and molecular events^4^. The most predictive health indicators related to these cellular and molecular events are the evaluation of the clinical parameters associated with the soft tissue and the bone level measurements on the radiographs^5^. As it is a dynamic organ, the stability of the surrounding bone is considered an important criterion to predict the prognosis of implant survival in the long-term^6^. In contrast, nicotine, which is the active ingredient involved in smoking, suppresses blood circulation in the bones and inhibits the normal functions of the bone forming cells^7^.

Even in smoking patients, since more than five decades, osseointegrated dental implants are being used to support prosthetic suprastructures associated with removable or fixed prostheses^8^. Although, the success of implant restorations is adversely affected by smoking and the resulting biological complications lead to the loss of the supporting bone, dental implant-supported fixed or removable prostheses are one of the most widely used treatment options^9^.

**Figure 1:**
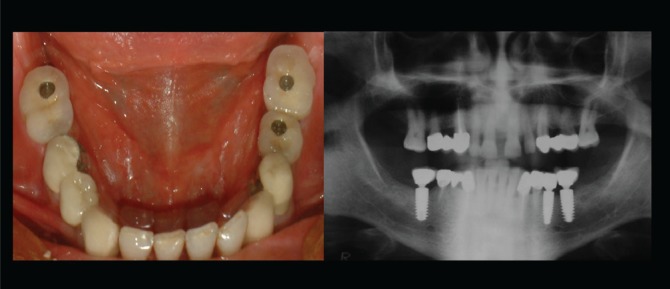
Implant supported fixed prostheses, missing teeth are replaced by directly cementing onto implants

**Figure 2:**
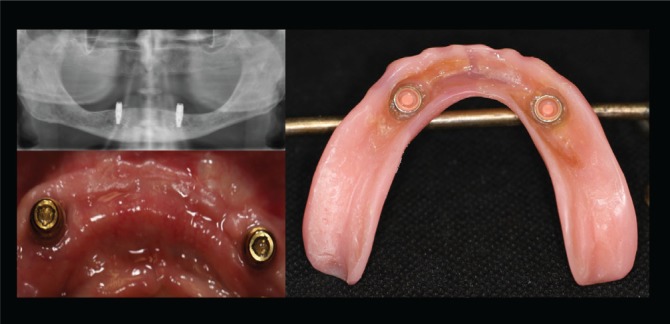
Implant supported removable prostheses, a prosthesis is not fixed and can be removed at any time

The choice between fixed or removable prostheses is a complex decision that requires many steps before recommending the treatment to patients. The clinician’s perspectives on the factors that affect the decision together with the expectations of the patient must be considered collectively since the types of prostheses involve different mechanisms^10^. Fixed prostheses involve replacing missing teeth by directly cementing them onto implants so that they can only be removed by a dentist (Figure 1), whereas removable prostheses can be taken out of the oral cavity by the patient^11,12^ (Figure 2). In the case of both types of prostheses, marginal bone loss (MBL) is analyzed since it is one of the original success criteria due to its potential effect that can lead to implant failure. The criteria of implant success in terms of MBL is a maximum MBL of 1 mm around the implant during the first year after placement; this has been considered as success in implant practice. Despite the variability in the literature, one year after the placement of a prosthesis, tissue stability is expected and an annual MBL of less than 0.2 mm is desired^10,11^.

There is no doubt about the potentially negative effects of smoking on implant treatment outcomes; however, there is a lack of understanding of the additional roles that different prostheses types play in patients who are smokers and non-smokers. The aim of this study was to analyze the effect of smoking in relation to the different types of dentures that might play key roles in the development of MBL. It is hypothesized that the type of dental prosthesis can alter the bone loss amount and that MBL rates are related to the anchoring systems of the dentures to the implants. The specific objective of this study was to examine whether smoking causes low or high MBL around implant-supported fixed or removable prostheses, after excluding other clinical factors.

## METHODS

### Study population

The study involved 138 patients (76 female, 62 male) aged 24–70 years (mean 55.53 ± 10.77 years) with 231 implants collectively. Among the 231 studied implants, 179 were placed in non-smokers while 52 were placed in smokers. Based on the type of prosthesis used, 137 of the implants supported fixed prostheses (in 62 males and 75 females, mean age 54.96 ± 12.24 years), while 94 supported removable prostheses (in 44 males and 50 females, mean age 56.17 ± 9.31 years). The regional ethics committee at the Eskisehir Osmangazi University, Turkey, approved (#25403353-050.99-E.15406) the study.

All the implants were randomly selected from the records of a private practice. The inclusion criteria were: patients with implant-supported fixed or removable prostheses. For the fixed prostheses, the missing teeth were treated with one-, two- or three-unit prostheses within the biomechanical force limits. *A priori* exclusion criteria included systemic bone disease e.g. Paget’s disease, or any parafunctional habits like bruxism which causes local bone problems. Additional exclusion criteria were presence of any acute or chronic sinus pathologies or other major systemic disease. Before the placement of implants, correct pre-operative periodontal diagnosis and the all-needed stabilisation were achieved. Following implant placement and prostheses placement, periodontal supportive therapy and regular maintenance appointments were scheduled. The periodontal health was the entire period stable without any signs of progression through bursts of activity or remission periods. The digital radiographs were taken on the day of implant placement and at 6, 12 and 24 months following functional loading with prostheses. The subjects who were engaged in this study were willing to enroll to receive check-ups during our recall periods.

After the implant’s loading, the patients were advised to attend to maintenance therapy at 6, 12 and 24 months by the dental practitioners and the authors. The entire recall periods comprised data collection, reevaluation, diagnosis, instrumentation, re-instruction and motivation for the awareness of risk factors and successful plaque control most importantly. Only the patients who attended all maintenance therapies were included in this study.

**Figure 3:**
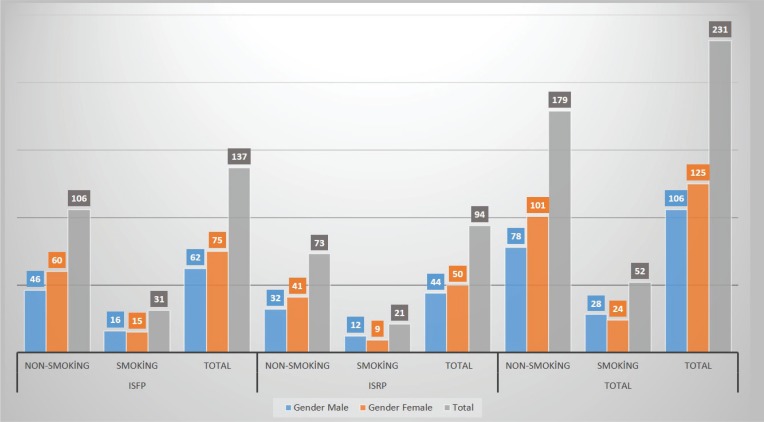
The evaluation of gender and prosthesis type distribution of implants in each group (ISFP: implant supported fixed prostheses. ISRP: implant supported removable prostheses)

MBL was analyzed on the mesial and distal sides of the implants. The first group comprised 94 implants (50 females and 44 males) with implant-supported removable prostheses (ISRPs). The second group comprised 137 implants (75 females and 62 males) with implant-supported fixed prostheses (ISFPs). Each group was divided into two groups: smokers (reported to have smoked a total of 100 cigarettes during their lifetime and currently smoked cigarettes) and non-smokers^13^. Figure 3 shows sociodemographic variables (age, sex, smoking status, used prostheses) while Tables 1, 2 and 3 show the clinical periodontal parameters of the study groups. All the patients were instructed on how to maintain oral health around the implants and remaining teeth, and educated on how to care for their prostheses. After placing the prostheses, baseline radiographs and clinical parameters were recorded. The patients were re-examined during each of the recall periods and new sets of radiographs together with clinical parameters were obtained.

**Table 1:** The influence of smoking on plaque index (PI)

*Plaque index*		*Smoking status*		
		*Smoking Mean±SD*	*Non-smoking Mean±SD*	*PC p*
**ISFP**	6 Month	0.66±0.48	0.60±0.55	<0.05
	12 Month	0.95±0.66	0.63±0.52	<0.05
	6 Month	0.66±0.48	0.60±0.55	<0.05
	24 Month	1.04±0.92	1.00±0.92	<0.05
	12 Month	0.95±0.66	0.63±0.52	<0.05
	24 Month	1.04±0.92	1.00±0.92	<0.05
**PC p**		6–12: <**0.05**	6–12: <**0.05**	0.165
		6–24: <**0.05**	6–24: <**0.05**	
		12–24: <**0.05**	12–24: <**0.05**	
**ISRP**	6 Month	0.51±0.67	0.59±0.56	<0.05
	12 Month	1.21±1.14	0.86±0.73	<0.05
	6 Month	0.51±0.67	0.59±0.56	<0.05
	24 Month	1.32±1.22	1.01±0.88	<0.05
	12 Month	1.21±1.14	0.86±0.73	<0.05
	24 Month	1.32±1.22	1.01±0.88	<0.05
**PC p**		6–12: <**0.05**	6–12: <**0.05**	0.242
		6–24: <**0.05**	6–24: <**0.05**	
		12–24: <**0.05**	12–24: <**0.05**	
**6 Month**	**ISFP**	0.66±0.48	0.60±0.55	<0.05
	**ISRP**	0.51±0.67	0.59±0.56	<0.05
	**PC p**	<**0.05**	>0.05	0.119
**12 Month**	**ISFP**	0.95±0.66	0.63±0.52	<0.05
	**ISRP**	1.21±1.14	0.86±0.73	<0.05
	**PC p**	<**0.05**	<**0.05**	0.004
**24 Month**	**ISFP**	1.04±0.92	1.00±0.92	<0.05
	**ISRP**	1.32±1.22	1.01±0.88	<0.05
	**PC p**	<**0.05**	>0.05	0.021

ISFP: Implant supported fixed prostheses (n=106 non-smokers, n=31 smokers). ISRP: Implant supported removable prostheses (n=73 non-smokers, n=21 smokers). Results are presented as mean and standard deviation (SD). Statistical significant at p<0.05. PC: pairwise comparisons.

**Table 2:** The influence of smoking on gingival index (GI)

*Gingival index*		*Smoking status*		
		*Smoking Mean±SD*	*Non-smoking Mean±SD*	*PC p*
**ISFP**	6 Month	0.29±0.26	0.39±0.51	<0.05
	12 Month	0.41±0.36	0.59±0.48	<0.05
	6 Month	0.29±0.26	0.39±0.51	<0.05
	24 Month	0.74±0.71	0.83±0.62	<0.05
	12 Month	0.41±0.36	0.59±0.48	<0.05
	24 Month	0.74±0.71	0.83±0.62	<0.05
**PC p**		6–12: <**0.05**	6–12: <**0.05**	0.102
		6–24: <**0.05**	6–24: <**0.05**	
		12–24: <**0.05**	12–24: <**0.05**	
**ISRP**	6 Month	0.37±0.27	0.44±0.29	<0.05
	12 Month	0.51±0.41	0.63±0.52	<0.05
	6 Month	0.37±0.27	0.44±029	<0.05
	24 Month	0.81±0.67	0.88±0.72	<0.05
	12 Month	0.51±0.41	0.63±0.52	<0.05
	24 Month	0.81±0.67	0.88±0.72	<0.05
**PC p**		6–12: <**0.05**	6–12: <**0.05**	0.091
		6–24: <**0.05**	6–24: <**0.05**	
		12–24: <**0.05**	12–24: <**0.05**	
**6 Month**	**ISFP**	0.29±0.26	0.39±0.51	<0.05
	**ISRP**	0.37±0.27	0.44±0.29	<0.05
	**PC p**	<**0.05**	<**0.05**	0.007
**12 Month**	**ISFP**	0.41±0.36	0.59±0.48	<0.05
	**ISRP**	0.51±0.41	0.63±0.52	<0.05
	**PC p**	<**0.05**	<**0.05**	0.006
**24 Month**	**ISFP**	0.74±0.71	0.83±0.62	<0.05
	**ISRP**	0.81±0.67	0.88±0.72	<0.05
	**PC p**	<**0.05**	<**0.05**	0.006

ISFP: Implant supported fixed prostheses (n=106 non-smokers, n=31 smokers). ISRP: Implant supported removable prostheses (n=73 non-smokers, n=21 smokers). Results are presented as mean and standard deviation (SD). Statistical significant at p<0.05. PC: pairwise comparisons.

**Table 3:** The influence of smoking on probing depth (PD)

*Probing depth*		*Smoking status*		
		*Smoking Mean±SD*	*Non-smoking Mean±SD*	*PC p*
**ISFP**	6 Month	3.19±0.40	3.18±0.29	>0.05
	12 Month	3.37±0.47	3.33±0.39	<0.05
	6 Month	3.19±0.40	3.18±0.29	>0.05
	24 Month	3.48±0.51	3.41±0.62	<0.05
	12 Month	3.37±0.47	3.33±0.39	<0.05
	24 Month	3.48±0.51	3.41±0.62	<0.05
**PC p**		6–12: <0.05	6–12: <0.05	0.032
		6–24: <0.05	6–24: <0.05	
		12–24: <0.05	**12–24: >0.05**	
**ISRP**	6 Month	3.17±0.27	3.17±0.39	>0.05
	12 Month	3.39±0.51	3.33±0.52	<0.05
	6 Month	3.17±0.27	3.17±0.39	>0.05
	24 Month	3.51±0.67	3.42±0.72	<0.05
	12 Month	3.39±0.51	3.33±0.52	<0.05
	24 Month	3.51±0.67	3.42±0.72	<0.05
**PC p**		6–12: <0.05	6–12: <0.05	0.164
		6–24: <0.05	6–24: <0.05	
		12–24: <0.05	12–24: <0.05	
**6 Month**	**ISFP**	3.19±0.40	3.18±0.29	>0.05
	**ISRP**	3.17±0.27	3.17±0.39	>0.05
	**PC p**	>0.05	>0.05	0.211
**12 Month**	**ISFP**	3.37±0.47	3.33±0.39	>0.05
	**ISRP**	3.31±0.51	3.33±0.52	>0.05
	**PC p**	>0.05	>0.05	0.235
**24 Month**	**ISFP**	3.48±0.51	3.41±0.62	<0.05
	**ISRP**	3.51±0.67	3.42±0.72	<0.05
	**PC p**	>0.05	>0.05	0.047

ISFP: Implant supported fixed prostheses (n=106 non-smokers, n=31 smokers). ISRP: Implant supported removable prostheses (n=73 non-smokers, n=21 smokers). Results are presented as mean and standard deviation (SD). Statistical significant at p<0.05. PC: pairwise comparisons.

The patients were informed several times about the importance of having a very thorough oral hygiene program at home and attending the regular recall visits at 6, 12 and 24 months. The routine and the same professionally administered peri-implant/ periodontal maintenance and prostheses check-ups were performed. At each routine recall visit specialists in the department of prosthodontics and department of periodontology documented the clinical data and patient history to maximize the lifespan of the implants. However, some of the patients did not attend scheduled recall appointments and were not counted as subjects of this study.

### Periodontal parameters

Clinical variables were analyzed to determine the effects of smoking on the health of the marginal areas around the implants. Full-mouth periodontal records of each patient were obtained. These records included the plaque index (PI)^14^, gingival index (GI)^15^, and probing depth (PD)^16^. All measurements were recorded on four different sites of each implant using a plastic probe with a force of 0.25 N (Hawe Click-Probe, KerrHawe SA, Bioggio, Switzerland) (Tables 1, 2 and 3).

The PI was recorded by moving the probe along the gingival margin of an implant. Visually, the presence of plaque was scored as follows: 0 = no plaque, 1 = plaque on probe, 2 = plaque on the implant seen by the naked eye, and 3 = abundance of soft matter. The GI was recorded 20 s after moving the periodontal probe along the gingival sulcus of an implant. Visually, the presence of bleeding was scored as follows: 0 = no bleeding, 1 = isolated bleeding spots visible, 2 = blood forms a confluent red line along the margin, and 3 = heavy or profuse bleeding. The PD (in mm) was determined by measuring the distance from a gingival margin to the base of the sulcus.

### Bone level measurements

**Figure 4:**
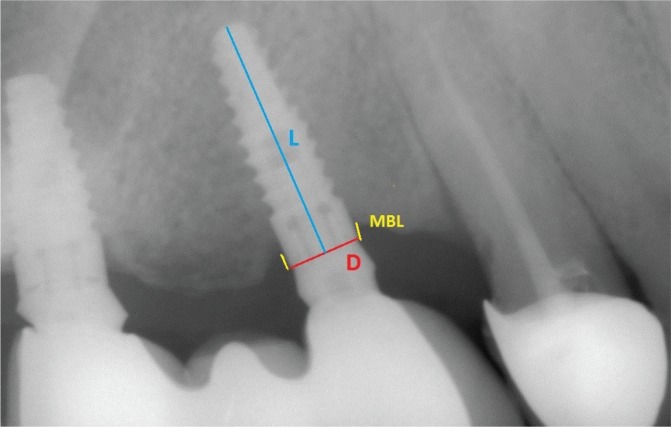
Measurement of marginal bone loss (D: diameter, L: length, MBL: marginal bone loss)

Panoramic radiographs (Planmeca, Proline XC, Helsinki, Finland) were taken immediately after prostheses placement and during every recall session. Mesial and distal marginal bone levels around all the implants were determined during the baseline and recall evaluations. As mentioned in the beginning, the images were scanned and digitized (Epson 1680 Pro, Seiko Epson Cooperation, Owa, Suwa, Nagano, Japan) and analyzed at ×20 magnification using a software program (CorelDraw 11.0; Corel Corp and Coral Ltd, Ottawa, Canada). According to the manufacturer’s instructions, the dimensions of the implants measured at the collar region were used as reference points. The distance from the widest part of the implant supracrestally to the level of the crestal bone was measured on the magnified images. The marginal bone levels were evaluated by comparing the bone levels measured at the time of prostheses placement with those measured during the follow-up periods. The distance between the reference point and the marginal bone level was recorded on each implant’s mesial and distal sites^17^ (Figure 4).

### Statistical analysis

Because we had different measurement time points, a general linear model was used in the statistical analyses methods. In this analysis we used a multiple approach. The collected data were analyzed using the SPSS Statistical Software version 21.0 (Armonk, NY: IBM Corp.). A Shapiro-Wilk test (p<0.05) revealed that the measurement scores were normally distributed. The influence of smoking on the MBL under different variables (age, gender) was analyzed using a 3-way analysis of variance (ANOVA) (One Factor Reputation). For the *post hoc* evaluation, the Bonferroni test was used. Possible correlations between the MBL levels and clinical periodontal measurements (PI, GI, and BOP) were computed by the Spearman ρ rank test. The results were assessed at a 95% confidence interval and at a significance level of 0.05.

## RESULTS

### Periodontal parameters

There were statistically significant differences between the fixed and removable implant- supported prostheses in terms of all the periodontal variables (Tables 1, 2 and 3). The state of oral hygiene was assessed via the PI measurements. When PI was evaluated in relation to tobacco consumption, significantly greater plaque amounts were found in the smokers among the patients with ISRPs at 12 and 24 months. The effect of smoking on the amount of plaque formed had increasingly significant consequences over time (Table 1). To assess the severity of inflammation among both the implant-supported fixed and removable prostheses users, the GI was examined and found to be more pronounced in non-smokers compared to that in smokers in a time-dependent manner (Table 2). In order to establish the state of health of the periodontal tissues, the PD was measured. When the PD was compared within 6 months of the placements, no differences were observed between smokers and non-smokers using both the implant-supported fixed or removable prostheses. However, at 12 and 24 months, a significant difference in PD was observed between smokers and non-smokers in both the implant-supported fixed and removable prostheses groups (p<0.05). Additionally, in the non-smoking group, no significant differences were observed between the PD values measured at 12 and 24 months (Table 3).

### Marginal bone loss

There were no significant correlations between the age (p>0.05) or the gender (p>0.05) of the patients and the marginal bone loss values in the present study. However, between time points, the marginal bones showed significant losses (p<0.05). Similar bone loss tendencies were observed on both the mesial (M) and distal (D) sites of the implants (Tables 4 and 5, respectively).

**Table 4:** Relation between mesial marginal bone loss (mean±SD in mm) and smoking

*Mesial marginal bone loss*		*Smoking status*		
		*Smoking Mean±SD*	*Non-smoking Mean±SD*	*PC p*
**ISFP**	6 Month	0.47±0.13	0.44±0.10	>0.05
	12 Month	0.82±0.18	0.80±0.13	<0.05
	6 Month	0.47±0.13	0.44±0.10	>0.05
	24 Month	0.93±0.17	0.88±0.16	<0.05
	12 Month	0.82±0.18	0.80±0.13	<0.05
	24 Month	0.93±0.17	0.88±0.16	<0.05
**PC p**		6–12: **<0.05**	6–12: <**0.05**	0.010
		6–24: <**0.05**	6–24: <**0.05**	
		12–24: <**0.05**	12–24: <**0.05**	
**ISRP**	6 Month	0.58±0.10	0.43±0.12	<0.05
	12 Month	0.91±0.22	0.88±0.13	<0.05
	6 Month	0.58±0.10	0.43±0.12	<0.05
	24 Month	1.11±0.22	0.86±0.24	<0.05
	12 Month	0.91±0.22	0.88±0.13	<0.05
	24 Month	1.11±0.22	0.86±0.24	<0.05
**PC p**		6–12: <**0.05**	6–12: <**0.05**	0.253
		6–24: <**0.05**	6–24: <**0.05**	
		12–24: <**0.05**	12–24: <**0.05**	
**6 Month**	**Fixed**	0.47±0.13	0.44±0.10	>0.05
	**Removable**	0.58±0.10	0.43±0.12	<0.05
	**PC p**	>0.05	>0.05	0.054
**12 Month**	**Fixed**	0.82±0.18	0.80±0.13	>0.05
	**Removable**	0.91±0.22	0.88±0.13	<0.05
	**PC p**	<**0.05**	>0.05	0.019
**24 Month**	**Fixed**	0.93±0.17	0.88±0.16	<0.05
	**Removable**	1.11±0.22	0.86±0.24	<0.05
	**PC p**	<**0.05**	>0.05	0.038

ISFP: Implant supported fixed prostheses (n=106 non-smokers, n=31 smokers). ISRP: Implant supported removable prostheses (n=73 non-smokers, n=21 smokers). Results are presented as mean and standard deviation (SD). Statistical significant at p<0.05. PC: pairwise comparisons.

**Table 5:** Relation between distal marginal bone loss (mean±SD in mm) and smoking

*Distal marginal bone loss*		*Smoking status*		
		*Smoking Mean±SD*	*Non-smoking Mean±SD*	*PC p*
**ISFP**	6 Month	0.52±0.14	0.45±0.11	>0.05
	12 Month	0.89±0.18	0.83±0.14	<0.05
	6 Month	0.52±0.14	0.45±0.11	>0.05
	24 Month	0.98±0.18	0.91±0.15	<0.05
	12 Month	0.89±0.18	0.83±0.14	<0.05
	24 Month	0.98±0.18	0.91±0.15	<0.05
**PC p**		6–12: <**0.05**	6–12: <**0.05**	0.049
		6–24: <**0.05**	6–24: <**0.05**	
		12–24: <**0.05**	12–24: <**0.05**	
**ISRP**	6 Month	0.61±0.06	0.45±0.13	<0.05
	12 Month	1.00±0.13	0.81±0.17	<0.05
	6 Month	0.61±0.06	0.45±0.13	<0.05
	24 Month	1.13±0.12	0.89±0.17	<0.05
	12 Month	1.00±0.13	0.81±0.17	<0.05
	24 Month	1.13±0.12	0.89±0.17	<0.05
**PC p**		6–12: <**0.05**	6–12: <**0.05**	0.827
		6–24: <**0.05**	6–24: <**0.05**	
		12–24: <**0.05**	12–24: <**0.05**	
**6 Month**	**Fixed**	0.52±0.14	0.45±0.11	<0.05
	**Removable**	0.61±0.06	0.45±0.13	<0.05
	**PC p**	>0.05	>0.05	0.048
**12 Month**	**Fixed**	0.89±0.18	0.83±0.14	<0.05
	**Removable**	1.00±0.13	0.81±0.17	<0.05
	**PC p**	<**0.05**	>0.05	0.049
**24 Month**	**Fixed**	0.98±0.18	0.91±0.15	<0.05
	**Removable**	1.13±0.12	0.89±0.17	<0.05
	**PC p**	<**0.05**	>0.05	0.024

ISFP: Implant supported fixed prostheses (n=106 non-smokers, n=31 smokers). ISRP: Implant supported removable prostheses (n=73 non-smokers, n=21 smokers). Results are presented as mean and standard deviation (SD). Statistical significant at p<0.05. PC: pairwise comparisons.

Concerning the smokers in the ISFP group, the MBL levels were 0.47±0.13 mmM and 0.52±0.14 mmD, 0.82±0.18 mmM and 0.89±0.18 mmD, and 0.93±0.17 mmM and 0.98±0.18 mmD at 6, 12 and 24 months, respectively. Further, concerning those in the ISRP group, the MBL levels were 0.58±0.10 mmM and 0.61±0.06 mmD, 0.91±0.22 mmM and 1.00±0.13 mmD, and 1.11±0.22 mmM and 1.13±0.12 mmD at 6, 12 and 24 months, respectively (Tables 4 and 5).

Concerning the non-smokers in the ISFP group, the MBL levels were 0.44±0.10 mmM and 0.45±0.11 mmD, 0.88±0.16 mmM and 0.83±0.14 mmD, and 0.88±0.16 mmM and 0.91±0.15 mmD at the 6, 12 and 24 months, respectively. Further, concerning those in the ISRP group, the MBL levels were 0.43±0.12 mmM and 0.45±0.13 mmD, 0.88±0.13 mmM and 0.81±0.17 mmD, and 0.86±0.24 mmM and 0.89±0.17 mmD at 6, 12 and 24 months, respectively (Tables 4 and 5).

At all time points, a significant time-dependent bone loss rate (p<0.05) was observed with both the implant-supported fixed or removable prostheses for smokers and non-smokers (Tables 4 and 5). When the MBL rates associated with the different sites of the implants were compared based on prosthesis type, significantly higher rates were observed only in the smokers from the ISRP group at 12 and 24 months (p<0.05) (Tables 4 and 5). For non-smokers, in both the ISRP and ISFP groups, similar bone loss amounts were observed with increased rates.

## DISCUSSION

It is of great interest to highlight whether the negative effects of smoking on the implant’s marginal bone level depend on the prostheses types. Via panoramic radiographs taken during the routine recall sessions, we found that smoking has a more destructive effect on MBL around dental implants supporting removable prostheses compared to around those supporting fixed prostheses. Since panoramic radiography has been reported to be a simple and reliable method of measuring bone level changes, we assessed the implant failure threats at the points where the bones were attached to the implants using recall session panoramic radiographs^17^. In accordance with the results of previous investigations, we confirmed that age and sex were not associated with MBL^18,19^.

Smoking has widespread systemic effects, many of which initiate mechanisms involved in poor responses to implant treatment^20^. In the present study, it was certainly expected that the tendency to develop MBL around implants increases in smokers^21^. However, the stage in the implant placement process at which a patient was included in our study was a crucial factor to consider. If we had selected our patients immediately after the placement of implants, the bone healing periods associated with the early or delayed wound healing processes might have been one of our main considerations to assess the effects of smoking^22,23^. We eliminated these important crucial time periods involved in osseointegration, which is known to be a time-dependent healing process. In the oral cavity, smoking causes reduced bone height and poor quality of bone healing, which leads to the loss of hard implant supporting tissue^23^. Although the detailed mechanisms by which smoking influences the osseointegration process remain unknown, generally, the failures of osseointegration depend on the deposition of fibrous tissue at the bone-implant interface. All these events are sensitive to the effects of nicotine^24^. Clinical studies have strongly suggested that smokers present a 1.69 times higher incidence of implant loosening compared to non-smokers during the first healing period before prostheses insertion. Furthermore, smoking has also been shown as a risk factor for delayed failures of implants, which might occur during the second stage of implant surgery^25,26^. However, in this retrospective study, we only analyzed implants that successfully survived in cases where all the implants had the same amount of marginal bone levels around the cervical collar regions at the time of prostheses insertion. In line with this baseline information, we are now able to eliminate the negative effects of smoking during the widely studied early or late wound healing stages^27^.

The soft tissue provides us with reliable information on the underlying pathology of the bone. The tissue that overlays the bone and surrounds the implants is structurally and functionally similar in some way to the tissue present around natural teeth. Compared to that of natural teeth, the soft tissue around dental implants is enlarged because of the longer junctional epithelium and the lower number of hemidesmosomal attachments^28-30^. Nicotine has a high diffusion potential and level of permeability through this weak gingival epithelium, which in turn causes a direct modulation of the osteoblastic activity underneath the epithelium. Thus, the higher MBL found in smoking groups is mostly related to the broken epithelial barrier^31,32^, which then leads to severely degraded connective tissue and utmost levels of MBL^33^.

From a clinical point of view, the attachments between the implants and prostheses must be thoroughly cleaned daily^10^. In our study, the lower amounts of MBL around the fixed prostheses of smokers might be due to the appropriate and efficient care of prostheses, such as cleaning and regular dentist controls, by the patients^34^. A fixed prosthesis offers benefits from both a functional and esthetic point of view and may be regarded as quite similar to a patient’s own natural dentition when compared to removable prostheses^10^. Because patients with fixed prostheses have reported enhanced social confidence and had the highest quality of life satisfaction scores^35^, most likely because they are more motivated to be more successful in their personal health care routines such as brushing their teeth regularly after smoking^36,37^. Furthermore, removable prostheses are more prone to plaque accumulation in the absence of effective oral hygiene. These prostheses must be taken out at nighttime and cleaned with a toothbrush; however, very often patients forget to remove them and sleep with them on^38^. It can be speculated that the cumulative effect of possible food remnants and the exposure to smoking creates a potential synergic effect that worsens the outcomes of the removable restorations, for which we observed a higher MBL in the patients. It is important to keep in mind that such an inflammatory effect is time dependent and that in the long-term, the pathological environment around the implant would have caused bacterial aggregation that activated immune mechanisms, finally leading to the highest amount of MBL, which was observed at 24 months^39^. Otherwise, in accordance with the results of previous studies, the bone loss for non-smokers was within the expected normal ranges: 1 mm in the first year and 0.2 mm in the following years^10,11^. Apart from these observations, the general opinion, according to which the distal region is difficult to reach and that performing oral hygiene is difficult in this region, was not observed in our study. The similar amounts of MBL on the mesial and distal sides of the implants could be a benefit derived from the careful motivation to achieve better oral health that was provided during each recall session.

According to the literature, independent of the periodontal conditions, the plaque control record is always significantly higher in smokers compared to that in non-smokers^40^. The cigarette smoke alters key periopathogenic surface molecules, which then enhances biofilm formation in the oral cavity^21^. When plaque control interventions are not implemented, wearing removable dental prostheses can be associated with a higher risk of developing periodontal disease^38,41,42^. However, the patients with implant treatment know the importance of being given detailed instructions on oral care and improve their oral health-related behaviors^43^. In accordance with previously published reports, the clinical periodontal parameters of our non-smoking patients (PI, GI, and PD) indicate that one prosthesis type is not superior to the other one. However, in the smoking group, the higher scores related to microbial biofilm formation (PI), increased bleeding (GI), and consequently increased pocket depth were the predictors of pathological developments in response to nicotine in the peri-implant sulcus^44^. Normally, the shallow crevice is bound apically by the coronal aspect of the epithelium and superiorly exits into the oral cavity with a fine balanced dynamic process^3^. Owing to the inhibition caused by nicotine, the balanced dynamics are disrupted, and the subsequent immune response causes the overproduction of pro-inflammatory cytokines. The increased cytokine levels are positively correlated with the PD values and breakdown of marginal bone^45^ around removable prostheses. Certainly, in relation to peri-implant problems, we always expect bleeding and consider increased bleeding as an initial sign indicating the development of a disorder. However, the masking effect of smoking owing to the direct vasoconstrictive effect of nicotine on blood vessels revealed diminished bleeding in the smoker group compared to that in the non-smoker group^44^.

Smoking has the strongest effect on promoting bacterial penetration into the deeper structures that acts concomitantly to change bacterial colonization^46^. Many authors have claimed that the direct effect of the heat of the smoke is a factor responsible for different oral mucosal disease^47^. However, the direct effect of smoke on MBL in the case of different types of prostheses has not been discussed. In our study, the design of removable prostheses might act as a barrier for tobacco smoke. The margins of the prostheses cover the alveolar region^10^, which creates an area in which the cigarette smoke is trapped. Because the smoke does not spread to other regions easily, the toxic radicals diffuse into the surrounding tissue^48^, which finally leads to the destruction of the bone-implant attachment^20^. In the ISFP group, the direct contact of the marginal area of the implant with the oral cavity provides the benefit of it being in contact with freely flowing saliva, which could clean toxins or substances around the margins^49^. Similarly, the effect of the higher temperatures associated with smoking on the sulcular areas is lowered by the saliva^50^. This is important because as the temperature changes, the physical and chemical properties of the subgingival ecosystem also change, and periodontal disease becomes more severe and MBL occurs^51^. It is critical to recognize that the progression of MBL is observed to be the slowest in the first two recall sessions, but it accelerates in the third recall. This means that being exposed to smoke first alters the plaque accumulation and bleeding, which then leads to bone loss, and the bone support becomes weaker over time^52^.

## CONCLUSIONS

Smoking hinders the survival rates of implants. However, appropriate oral hygiene instructions with well controlled recall periods increase the strength of the surrounding bone. Further, in the literature, the detailed information regarding the interaction between smoking and implant prosthesis designs has explanatory gaps. By better understanding reasons behind MBL, it will be possible to plan tailor-made treatments due to patient diversity. The current results presumably indicate that MBL rates are less in the case of the removable design in smokers compared to those in non-smokers. This observation can shed light on the mutual roles that dental prostheses and smoking play on activating biological effects; this can be used as a reference for further studies and consequently aid in reducing MBL.
